# A prospective observational study on the value of HPV E6/E7 oncoprotein immunocytochemistry for triaging HPV-positive women

**DOI:** 10.3389/fmed.2026.1745006

**Published:** 2026-04-01

**Authors:** Zangyu Pan, Qi Sun, Yixiao Wang, Jiamin Zhang, Yiqun Zhang, Jinwei Miao

**Affiliations:** 1 Department of Gynecologic Oncology, Beijing Obstetrics and Gynecology Hospital, Capital Medical University, Beijing Maternal and Child Health Care Hospital, Beijing, China; 2 Laboratory for Clinical Medicine, Capital Medical University, Beijing, China; 3 Department of Obstetrics and Gynecology, Taihe Hospital, Shiyan, China

**Keywords:** clinical diagnosis and triage, exfoliated cervical cells, HPV E6/E7 oncoprotein, human papillomavirus, immunocytochemical staining

## Abstract

**Background:**

While human papillomavirus testing has become the primary screening modality for cervical cancer prevention, its implementation necessitates supplementary triage strategies. The viral oncoproteins E6 and E7, serving as primary mediators of high-risk HPV (hrHPV)-induced carcinogenesis, emerge as viable candidates for this risk stratification. We aimed to examine the clinical diagnostic performance of HPV E6/E7 immunocytochemical (ICC) assay in exfoliated cervical cells samples, to investigate the feasibility of using it as a potential biomarker in clinical triage algorithms for hrHPV-positive populations.

**Methods:**

We prospectively collected and analyzed 1,005 hrHPV-positive cases from Beijing Obstetrics and Gynecology Hospital, Capital Medical University during September 2023 and March 2025. The diagnostic and triaging performance of HPV E6/E7 ICC assay were evaluated by sensitivity, specificity, positive predictive values, negative predictive values and operating characteristic curve.

**Results:**

When used to detect CIN2+, the sensitivity of HPV E6/E7 ICC detection is (0.88, 95% CI: 0.82–0.92), which is better than that of cytological detection (0.609, 95% CI: 0.53–0.67), *χ*^2^ = 26.23, *p <* 0.05; the NPV of. HPV E6/E7 ICC detection is (0.95, 95% CI: 0.92–0.97), which is better than that of cytological detection (0.85, 95% CI: 0.82–0.88), *χ*^2^ = 38.14, *p <* 0.05. When the HPV typing test result is 16/18+,the sensitivity of HPV E6/E7 ICC detection is (0.93, 95% CI:0.86–0.96), which is better than that of cytological detection (0.61, 95% CI:0.52–0.70), *χ*^2^ = 33.47, *p <* 0.05; the NPV of HPV E6/E7 ICC detection is (0.940, 95% CI:0.89–0.97), which is better than that of cytological detection (0.81, 95% CI:0.75–0.85), *χ*^2^ = 13.84, *p <* 0.05. When the HPV typing test result is other hrHPV type, the sensitivity of HPV E6/E7 ICC detection is (0.81, 95% CI:0.71–0.89), which is better than that of cytological detection (0.75, 95% CI:0.64–0.84), *χ*^2^ = 0.914, *p* = 0.339; the NPV of HPV E6/E7 ICC detection is (0.95, 95% CI:0.92–0.97), which is better than that of cytological detection (0.91, 95% CI:0.86–0.94), *χ*^2^ = 4.29, *p* = 0.038.

**Conclusion:**

HPV E6/E7 ICC assay with high-sensitivity feature may be potential new biomarkers for hrHPV positive women. In addition, its triaging performance is better than that of cytology.

## Introduction

According to GLOBCAN 2022 report, cervical cancer (CC) ranks as the fourth most prevalent malignancy globally in terms of both incidence and mortality among women (1). Population-level analyses estimate approximately 661,000 new cervical cancer cases and 348,000 related deaths worldwide in 2022, demonstrating a notable increase from 2020 figures (1, 2). The World Health Organization (WHO) CC elimination strategy, which combines human papillomavirus (HPV) vaccination programs, early-stage cytological/virological screening, and standardized treatment of precancerous lesions, has positioned several high-income countries on course to achieve disease elimination milestones by 2030 (3).

The established pathophysiological consensus identifies persistent high-risk HPV (hrHPV) infection as the principal etiologic driver in >90% of cervical carcinomas (4). Substantial evidence confirms that persistent hrHPV infection serves as an essential etiological factor for cervical carcinogenesis (5). However, the natural history of cervical carcinogenesis follows a protracted timeline, typically spanning 5–10 years from initial hrHPV exposure to invasive carcinoma development (6), and the clinical implementation of hrHPV testing faces challenges: the disproportionally higher population prevalence of transient hrHPV infections relative to histologically confirmed high-grade squamous intraepithelial lesion (HSIL) substantially diminishes the positive predictive value of standalone virological screening (7).

Actually, direct colposcopy referral of women with positive results for several situations of HPV genotyping test has become a clinical algorithm in some HPV screening guidelines (8). Nevertheless, the main shortcoming of HPV genotyping test is that we cannot distinguish between transient and persistent infections. Over 90% of infections due to hrHPV will be cleared spontaneously after 2 years (9). This necessitates the development of validated triage strategies to accurately stratify hrHPV-positive individuals, balancing timely intervention against risks of overdiagnosis and avoiding excessive overtreatment that causes infrastructural, financial, health and psychological burden (10, 11).

The benefits of hrHPV-based screening can be enhanced by the application of triage tests (12). The E6 and E7 oncoproteins encoded by hrHPV genotypes function as primary regulators of viral persistence and tumorigenesis (13). E6 interferes with the regulation of cell-cycle control and apoptosis, inactivating the p53 tumor suppressor gene; E7 protein destroys cell cycle regulation by binding and inactivating pRb, resulting in abnormal cell proliferation (6). Therefore, the detection of this virus cancer protein can provide a more specific method to detect precancerous lesions and cancer.

Previous studies have confirmed the performance of HPV E6/E7 mRNA as a biomarker of validated triage strategies (14, 15). Immunocytochemistry detects abnormal expression of HPV E6/E7 oncoprotein in exfoliated cervical cells (15), which has the advantages of non-invasive, simple and economical. In this study, we evaluated the clinical efficacy of immunocytochemistry (ICC) for E6 and E7 oncoproteins (E6-ICC and E7-ICC). This method, referred to herein as the HPV E6/E7 oncoprotein immunocytochemical test (HPV E6/E7 ICC assay), was applied to liquid-based cytology samples.

## Materials and methods

### Study population

**Figure 1:**
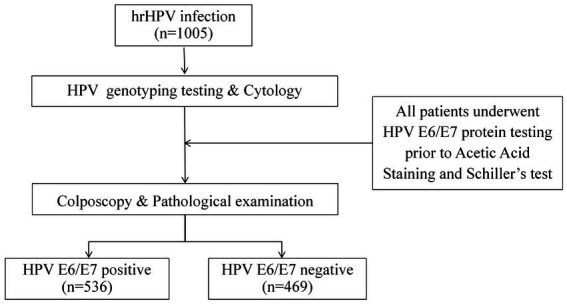
Flowchart of participant inclusion in the evaluation of the HPV E6/E7 ICC assay.

This prospective cross-sectional study recruited HPV-positive women at Beijing Obstetrics and Gynecology Hospital between September 2023 and March 2025. Inclusion criteria were (1) 25–65 years old females; (2) women with available hrHPV genotyping and liquid-based cytology (LBC) results scheduled for colposcopy referral; (3) no recorded severe organ dysfunction/mental illnesses; (4) no CC, hysterectomy, conization of the uterine cervix or pelvic radiation therapy history; and (5) voluntary participation and a demonstrable ability to provide medical information. Exclusion criteria included pregnancy, lactation, inadequately documented medical records, participants who dropped out prematurely, and engagement in activities including sexual intercourse, vaginal washing, or vaginal medication within 48 h prior to sampling. Flowchart of participant inclusion in the evaluation of the HPV E6/E7 protein testing was shown in Figure 1.

To inprove verification bias mitigation procedures, we use adjudication blinding protocol: ICC assay interpretation was performed by technicians masked to all clinical data including HPV genotyping, cytology results, and final histology diagnoses. Conversely, pathologists evaluating biopsy specimens were blinded to ICC results.

### Liquid-based cytology (LBC) examination

Exfoliated cervical cells collected by Cytobrush were suspended in PreservCyt collection medium (Hologic Inc., Marlborough, MA, USA) for LBC analysis All specimens were first processed via ThinPrep^®^ cytology system (Hologic Inc.). Subsequently, the resulting LBC slides were evaluated according to the Bethesda System (TBS, 2014) (16). According to TBS, cytological morphology is classified as negative for intraepithelial lesion or malignancy (NILM), atypical squamous cells of undetermined significance (ASC-US), atypical glandular cells (AGC), low-grade squamous intraepithelial lesion (LSIL), atypical squamous cells cannot exclude high-grade squamous intraepithelial lesion (ASC-H), HSIL, squamous cell carcinoma (SCC), as well as adenocarcinoma (AD).

### HPV genotyping testing

HPV genotyping was performed on cervical specimens collected with the Female Sample Collection Kit (Hybribio) during clinical examination and preserved in 3.5 mL of solution after routine cytology. HPV genotyping was conducted on an ABI 7500 Real-Time PCR System (Thermo Fisher Scientific, USA) using the HBRT- 23 HPV Genotyping Real-time PCR Kit (HybriBio Ltd., China; Certificate No. 20153401700), with DNA extracted from the specimens according to the manufacturer’s instructions. The genotyping assay targeted 14 high-risk HPV (hrHPV) types (16, 18, 31, 33, 35, 39, 45, 51, 52, 56, 58, 59, 66, and 68), along with several low-risk/other types (6, 11, 42, 43, 44, 53, 73, 81, and 82). An internal cellular control was included in each sample to monitor the entire testing process from DNA extraction to amplicon detection (17). Subsequent analysis was restricted to samples that tested positive for any of the 14 targeted hrHPV genotypes. Current CC screening guidelines (18–20) recommend immediate colposcopy referral for individuals positive for HPV 16/18. Based on this, we adopted an operational definition for our analysis: cases with HPV 16/18 were classified as “positive,” and those with other hrHPV genotypes were classified as “negative” in the analysis.

### HPVE6/E7 liquid-based cytology sample collection

The HPVE6/E7 LBC samples were collected before acetic acid test in the colposcopy room. The management of abnormal screening test results was based on the Chinese guidelines (18), with the extension to American Society for Colposcopy and Cervical Pathology 2012 and 2015 guidelines for cases not covered by the Chinese guidelines (19, 20). Following collection from the endocervical canal and cervical surface with a specialized cervical brush (Shenzhen MandeLab Co., Ltd.) via 2–3 clockwise rotations, the clinical specimens of exfoliated cervical epithelial cells were stored in Attogen LBC preservation solution (Cat. No. C075; Attogen, Suzhou, China). Upon collection, all samples were stored at 4 °C and all assays were completed within 14 days.

### HPVE6/E7 immunocytochemistry

Immunostaining for E6 and E7 oncoproteins was conducted on LBC samples. The staining was performed automatically using an AttoStar 4800A stainer (Attogen) with specific E6-ICC and E7-ICC antibody kits (Attogen), respectively, following the manufacturer’s instructions and established methods as previously reported (21, 22). The automated immunohistochemical staining procedure comprised sequential steps of antigen retrieval, incubation with primary antibodies, incubation with horseradish peroxidase (HRP)-conjugated secondary antibodies, and finally chromogenic development using 3,3′-diaminobenzidine (DAB) with hematoxylin counterstaining. Standard control slides were included to calibrate the instrumentation and immunoreagents for each E6-ICC and E7-ICC assay run, in accordance with the manufacturer’s protocols. The following cervical cancer cell lines, obtained from the American Type Culture Collection (ATCC, USA), served as controls: CaSki (HPV16 positive, CRL-1550™), HeLa (HPV18 positive, CCL-2™), and C-33A (HPV negative, HTB-31™). After the automated staining was complete, the slides were cover slipped. Staining results were evaluated by optical microscopy using 20x and 40x objective lenses (Olympus Corporation, Tokyo, Japan).

### Optimized immunohistochemistry protocol

Pre-Antigen Retrieval: Samples were hydrated in 50% ethanol (10 min), followed by two 3-min deionized water washes. Antigen Retrieval: Samples in Tris-EDTA buffer (pH 9.0) were heated to 95–99 °C (10 min) in a water bath, then cooled to room temperature. Washed twice with PBST (3 min each). Endogenous Peroxidase Blocking: Tissue sections were circled with an IHC pen, incubated with peroxidase blocking reagent (10 min), and washed twice with PBST (3 min each). Primary Antibody Incubation: Anti-HPV E7 antibody (150 μL) was applied (30 min, RT), followed by four PBST washes (3 min each). Secondary Antibody Incubation: HRP-conjugated goat anti-mouse IgG (2–3 drops) was added (30 min, RT), washed four times with PBST (3 min each). DAB Development: Fresh DAB substrate (100 μL) was applied (≤10 min, microscopically monitored). Reaction stopped by two 2-min deionized water rinses. Hematoxylin Counterstaining: Sections were stained with hematoxylin (10–30 s), rinsed, and blued in tap water (10 min). Dehydration & Mounting: Sequential dehydration in 70% → 85% → 95% → 100% ethanol (3 min each), xylene clearance (5 min), and neutral resin mounting. E6 protein staining is the same as above.

Staining results were interpreted by three qualified cytological pathologists. Kappa statistic was used for inter-observer agreement (Kappa = 0.976*, p <* 0.05). An evaluation form was used to capture data from each E6 or E7 immunostaining test. This original data form included the information such as number of stained cells, and types of cells exhibited staining, i.e., dysplastic, atypical, metaplastic, endocervical. The cervical squamous epithelial lesion cells were stained with HPV-E6 antibody, and the staining was localized as nucleus and cytoplasm, and the staining intensity was moderate to deep brown staining. The HPV-E7 antibody-stained cervical squamous epithelial lesion cells, which were located as cytoplasm, and the cytoplasm was obviously brown stained, and the nucleus was usually blue (pine fine staining). The interpretation of staining results is based on the expression mechanism of E6/E7 cancer protein and the immune response principle of antibody detection, and the positive staining is distinguished. The standard of cells is based on the principle of “dyeing quality/intensity/location/cell loading”. There is more than one positive infection in the cut. Cell samples, the test result is “positive”. E6 positive definition: squamous epithelial cells in the surface or middle layer of cervical epithelium, with moderate to strong staining intensity; Dyeing localization both the nucleus and cytoplasm are colored. E7 positive definition: squamous epithelial cells in the surface or middle layer of cervical epithelium, with moderate to strong staining intensity; dyeing localization cytoplasmic staining. Exclusion criteria: cut off impurities, pollutants and micro-infections; Upper cells (cervical canal, culture, etc.) and cell clusters or accumulation lead to specific characteristics.

**Figure 2:**
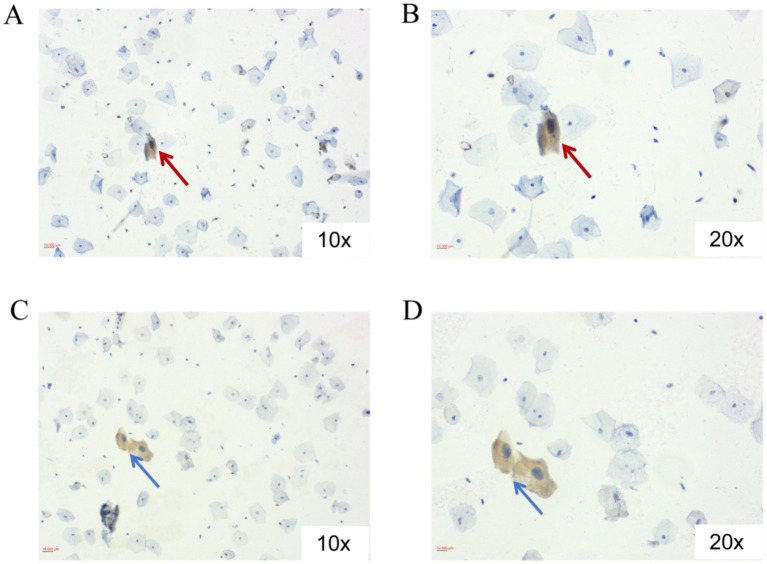
Positive HPV E6/E7 immunocytochemistry results. **(A)** HPV-E6 positive cervical squamous epithelial cells (red arrow), 10×; **(B)** HPV-E6 positive cervical squamous epithelial cells (red arrow), 20×; **(C)** HPV-E7 positive cervical squamous epithelial cells (blue arrow), 10×; **(D)** HPV-E7 positive cervical squamous epithelial cells (blue arrow), 20×.

A positive test result was judged by one or more positively stained cells. If the number of cervical epithelial cells on the slide does not meet more than 5,000, but the staining result has one or more positively stained cells, the sample is considered as a “positive” result. If the number of cervical epithelial cells is less than 5,000 and no positively stained cells were observed, it is considered an invalid sample and cannot be interpreted as a “negative” result. In the same sample, if E6 or E7 is positive, it is finally judged as a positive result. Positive immunostaining results (Figure 2) were independently assessed by two senior cytopathologists in a blinded manner. Any discrepant assessments were resolved by a third pathologist to establish a consensus diagnosis.

### Colposcopy and biopsy

All colposcopies were performed by highly trained personnel. All participants underwent colposcopy examination, and colposcopy-directed biopsies were performed on visible lesions or 3–4 random biopsies were taken from the normal-appearing cervix, unless the patient refused to undergo a biopsy. For women with TZ3, endocervical curettage (ECC) was performed. Two senior pathologists independently evaluated the pathological biopsy results. In cases of discrepancy between the two pathologists, a third pathologist was consulted to reach a consensus diagnosis. Biopsy results were categorized as follows: cervicitis/metaplasia, cervical intraepithelial neoplasia grade 1–3 (CIN1-3), carcinoma *in situ* (CIS), and invasive carcinoma. A diagnosis of HSIL or worse (HSIL+) was established based on positive p16 and Ki-67 immunohistochemistry staining. Abnormal results were treated according to standard guidelines.

### Data analyses

Two researchers collected all the data and entered it into a Microsoft Excel sheet. Data analysis was performed using SPSS software version 26.0. Statistical significance was set at *p* ≤ 0.05. And 95% confidence intervals (CI) was calculated. Absolute estimates and 95% CI for sensitivity, specificity, positive predictive values (PPV), and negative predictive values (NPV) were also calculated. McNemar tests were used to compare the detection rate. Area under the receiver operating characteristic curve (ROC) values were calculated using standard definitions. We used Pearson’s chi-squared tests to compare sensitivity, specificity, PPV and NPV.

## Results

In Table 1, the analysis of 1,005 hrHPV positive cases yielded the following results: histologically confirmed CIN2 + and CIN3 + were found in 20.1% (202/1005) and 9.7% (97/1005) of cases; cytological abnormalities (≥ASC-US) were present in 54.4% (546/1005) of women, respectively. Overall, 41.3% (415) of cases tested positive for HPV 16/18 (including co-infections), and 58.7% (590) had other hrHPV types (non-HPV16/18). The overall positivity rate for HPV E6/E7 ICC assay was 53.4% (536/1005).

**Table 1:** Baseline characteristics of the study cohort.

Items	*N*	(%)
Liquid-based cytology		
- NILM	459	(45.6)
- ASC-US	277	(27.6)
- ASC-H	22	(2.2)
- LSIL	178	(17.7)
- HSIL	51	(5.1)
- AGC	10	(1.0)
- SCC	8	(0.8)
HPV E6/E7 ICC assay		
- Positive	536	(53.3)
- Negative	469	(46.7)
hrHPV testing		
- HPV 16/18 (+)	415	(41.3)
- HPV 16/18 (−)	590	(58.7)
Histology diagnosis		
- Cervicitis/ metaplasia	485	(48.3)
- CIN1	318	(31.6)
- CIN2	105	(10.4)
- CIN3	75	(7.5)
- Invasive carcinoma/Carcinoma *in situ*	22	(2.2)

N, number; HPV, human papillomavirus; NILM, no intraepithelial lesion or malignancy; ASC-US, atypical squamous cells of undetermined significance; ASC-H, atypical squamous cells that cannot exclude high-grade squamous intraepithelial lesion; LSIL, low-grade squamous intraepithelial lesions; HSIL, high-grade squamous intraepithelial lesions; AGC, atypical glandular cells; SCC, squamous cell carcinoma; CIN, cervical intraepithelial neoplasia; CIN1, CIN grade 1.

The HPV E6/E7 ICC assay demonstrated higher HSIL+ detection sensitivity than HPV 16/18 genotyping, with rates of 88.1% vs. 60.4% at the CIN2 + threshold and 88.0% vs. 65.6% at the CIN3 + threshold (Table 2). Furthermore, the agreement between the HPV E6/E7 ICC assay and cytology in detecting HSIL+ was poor, with kappa values of 0.081 for CIN2 + and 0.166 for CIN3+. The discordance was statistically significant (McNemar test, *p <* 0.05 for CIN2 + and *p* = 0.023 for CIN3%), particularly notable in these clinically critical patient groups (Table 3).

**Table 2:** Concordance between the HPV E6/E7 ICC Assay and HPV-16/18 for the detection of high-grade cervical lesions (CIN2 + and CIN3+).

Analysis	Items	HPV E6/E7 ICC assay			Kappa index (95% CI)	McNemar test (*p*-value)
		HPVE6/E7 (+)	HPVE6/E7 (−)	Total		
All cases	hrHPV testing				0.164 (0.11–0.22)	*<*0.050
	HPV 16/18 (+)	263	152	415		
	HPV 16/18 (−)	273	317	590		
	Total	536	469	1,005		
CIN2+	hrHPV testing				0.129 (0.02–0.24)	*<*0.050
	HPV 16/18 (+)	113	9	122		
	HPV 16/18 (−)	65	15	80		
	Total	178	24	202		
CIN3+	hrHPV testing				0.142 (−0.04–0.032)	*<*0.050
	HPV 16/18 (+)	61	5	66		
	HPV 16/18 (−)	25	6	31		
	Total	68	11	97		

CI, confidence interval; HPV, human papillomavirus; CIN, cervical intraepithelial neoplasia.

**Table 3:** Concordance between the HPV E6/E7 ICC assay and cytology for the detection of high-grade cervical lesions (CIN2 + and CIN3+).

Analysis	Items	HPV E6/E7 ICC assay			Kappa index (95% CI)	McNemar test (*p*-value)
		HPVE6/E7 (+)	HPVE6/E7 (−)	Total		
All cases	Cytology				0.155(0.09–0.22)	0.661
	≥ASCUS	330	216	546		
	*<*ASCUS	206	253	459		
	Total	536	469	1,005		
CIN2+	Cytology			Total	0.081(−0.04–0.20)	*<*0.050
	≥ASCUS	122	13	135		
	*<*ASCUS	56	11	67		
	Total	178	24	202		
CIN3+	Cytology			Total	0.166(0.00–0.37)	0.023
	≥ASCUS	68	6	74		
	*<*ASCUS	18	5	23		
	Total	86	11	97		

CI, confidence interval; ASC-US, atypical squamous cells of undetermined significance; HPV, human papillomavirus; CIN, cervical intraepithelial neoplasia.

**Figure 3:**
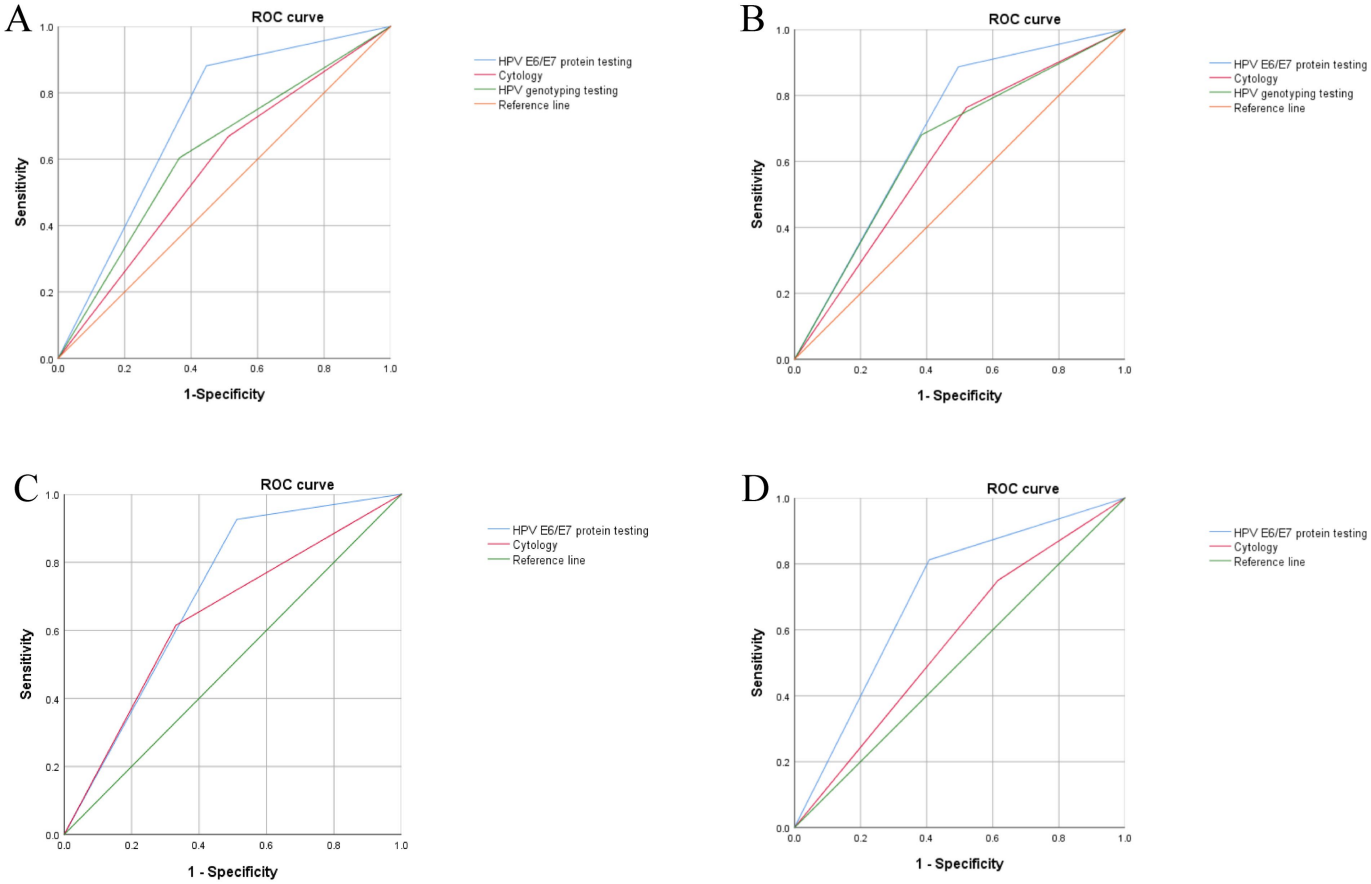
Receiver operating characteristic (ROC) curves of different detection assays. **(A)** Performance of HPVE6/E7 protein testing, HPV genotyping testing, and liquid-based cytology for the detection of CIN2+. AUC (95% CI) for HPV E6/E7: 0.718 (0.68–0.75), AUC (95% CI) for HPV genotyping testing: 0.620 (0.58–0.66), AUC (95% CI) for cytology: 0.578 (0.54–0.62); **(B)** Performance of HPVE6/E7 protein testing, HPV genotyping testing, and liquid-based cytology for the detection of CIN3+. AUC (95% CI) for HPV E6/E7: 0.696 (0.65–0.74), AUC (95% CI) for HPV genotyping testing: 0.648 (0.59–0.71), AUC (95% CI) for cytology: 0.648 (0.59–0.71); **(C)** Performance of different strategies for the detection of CIN2 + in HPV16/18 + women. AUC (95% CI) for HPV E6/E7: 0.94 (0.89–0.97), AUC (95% CI) for cytology: 0.81 (0.75–0.85); **(D)** Performance of different strategies for the detection of CIN2 + in HPV16/18- women. AUC (95% CI) for HPV E6/E7: 0.95 (0.92–0.97), AUC (95% CI) for cytology: 0.91 (0.86–0.94).

For discriminating HSIL+, the HPV E6/E7 ICC assay demonstrated superior performance to the strategies recommended in current screening guidelines [HPV 16/18 genotyping and cytology (ASC-US+)], with corresponding area under curves (AUCs) of 0.718, 0.620, and 0.578 for CIN2 + (*p <* 0.05) and 0.696, 0.648, and 0.622 for CIN3 + (*p <* 0.05) in Figure 3 and Table 4. These performance metrics underscore the assay’s significant clinical value as a triage tool for hrHPV-positive women. Moreover, this assay achieves the highest sensitivity for CIN2 + (88%) and CIN3 + (87%), substantially reducing missed diagnoses, and an NPV of 97% for CIN3+, thus offering an optimal balance for triage use in Table 4.

**Table 4:** Diagnostic performance for CIN2 + and CIN3 + in different tests.

Criteria	Sensitivity (95% CI)	Specificity (95% CI)	PPV (95% CI)	NPV (95% CI)	AUC (95% CI)
CIN2+					
- HPV E6/E7 ICC assay	0.88 (0.82–0.92)	0.55 (0.52–0.59)	0.33 (0.29–0.37)	0.95 (0.92–0.97)	0.718 (0.68–0.75)
- HPV genotyping testing	0.60 (0.53–0.67)	0.64 (0.60–0.67)	0.29 (0.25–0.34)	0.86 (0.83–0.89)	0.620 (0.58–0.66)
- Cytology	0.67 (0.60–0.73)	0.49 (0.45–0.52)	0.25 (0.21–0.29)	0.85 (0.82–0.88)	0.578 (0.54–0.62)
CIN3+					
- HPV E6/E7 ICC assay	0.87 (0.80–0.94)	0.50 (0.47–0.54)	0.16 (0.13–0.19)	0.97 (0.96–0.99)	0.696 (0.65–0.74)
- HPV genotyping testing	0.68 (0.58–0.77)	0.62 (0.58–0.65)	0.16 (0.13–0.20)	0.95 (0.93–0.96)	0.648 (0.59–0.71)
- Cytology	0.76 (0.66–0.84)	0.48 (0.45–0.51)	0.14 (0.11–0.17)	0.95 (0.92–0.97)	0.622 (0.57–0.68)

CI, confidence interval; PPV, positive predictive values; NPV, negative predictive values; AUC, area under ROC curve.

HPV 16/18 typing positive means HPV genotyping testing positive results.

Sensitivity (E6/E7 vs. Cytology): *χ*^2^ = 26.23, *p* < 0.05; Specificity (E6/E7 vs. Cytology): *χ*^2^ = 7.01, *p* = 0.008; PPV (E6/E7 vs. Cytology): *χ*^2^ = 9.47, *p* = 0.002; NPV (E6/E7 vs. Cytology): *χ*^2^ = 38.14, *p* < 0.05. Sensitivity (E6/E7 vs. 16/18): *χ*^2^ = 40.61, *p* < 0.05; Specificity (E6/E7 vs. 16/18): *χ*^2^ = 10.91, *p* = 0.001; PPV (E6/E7 vs. 16/18): *χ*^2^ = 1.57, *p* = 0.21; NPV (E6/E7 vs. 16/18): *χ*^2^ = 21.03, *p* < 0.05. AUC (HPV E6/E7 protein testing) *p* < 0.001; AUC (HPV genotyping testing) *p* = 0.001; AUC (Cytology) *p* < 0.05.

Sensitivity (E6/E7 VS. Cytology):*χ*^2^ = 5.14, *p* = 0.023; Specificity (E6/E7 VS. Cytology): *χ*^2^ = 1.07, *p* = 0.302; PPV (E6/E7 VS. Cytology): *χ*^2^ = 1.33, *p* = 0.248; NPV (E6/E7 VS. Cytology): *χ*^2^ = 4.67, *p* = 0.031; Sensitivity (E6/E7 VS. 16/18): *χ*^2^ = 12.16, *p* < 0.05; Specificity (E6/E7 VS. 16/18): *χ*^2^ = 23.80, *p* < 0.05; PPV (E6/E7 VS. 16/18): *χ*^2^ = 0.003, *p* = 0.953; NPV (E6/E7 VS. 16/18): *χ*^2^ = 5.81, *p* = 0.016. AUC (HPV E6/E7 protein testing) *p* < 0.05; AUC (HPV genotyping testing) *p* < 0.001; AUC (Cytology) *p* < 0.05.

The HPV E6/E7 ICC assay demonstrated a lower false-negative rate (FNR; 1 − sensitivity) for CIN2 + than both cytology and HPV 16/18 genotyping (12% vs. 33 and 40%, respectively; *p <* 0.05). This advantage was also observed for CIN3+, with an FNR of 13% compared to 24% for cytology and 32% for HPV 16/18 testing. Although high sensitivity in a triage test often comes at the cost of specificity, the specificity of the E6/E7 ICC assay remained comparable to that of cytology. Moreover, its positive predictive value (PPV) for CIN2 + was superior to that of the other tests (33% [95% CI: 0.29–0.37]), supporting its clinical utility in more accurately identifying HPV-positive women who require further intervention (Table 4).

Nevertheless, the HPV E6/E7 ICC assay maintained superior NPV performance in clinical differentiation. Compared to cytology (≥ASCUS), the HPV E6/E7 ICC assay showed substantially higher sensitivity (93% vs. 61%) and NPV (94% vs. 81%) for HSIL+ detection among HPV 16/18-positive individuals. This advantage in sensitivity (81% vs. 75%) was likewise observed in the other hrHPV (non-HPV16/18) group (Table 5).

**Table 5:** Diagnostic performance of triage strategies for CIN2 + in women positive for high-risk HPV.

HPV genotyping testing	Strategies	Sensitivity (95% CI)	Specificity (95% CI)	PPV (95% CI)	NPV (95% CI)	AUC (95% CI)
HPV16/18+	HPV E6/E7 protein testing	0.93 (0.86–0.96)	0.49 (0.43–0.55)	0.43 (0.37–0.49)	0.94 (0.89–0.97)	0.707 (0.66–0.76)
	Cytology (≥ASCUS)	0.61 (0.52–0.70)	0.67 (0.61–0.72)	0.44 (0.36–0.51)	0.81 (0.75–0.85)	0.642 (0.58–0.70)
HPV16/18−	HPV E6/E7 protein testing	0.81 (0.71–0.89)	0.59 (0.55–0.63)	0.24 (0.19–0.29)	0.95 (0.92–0.97)	0.702 (0.65–0.76)
	Cytology (≥ASCUS)	0.75 (0.64–0.84)	0.38 (0.34–0.43)	0.16 (0.13–0.20)	0.91 (0.86–0.94)	0.567 (0.50–0.63)

CI, confidence interval; PPV, positive predictive values; NPV, negative predictive values; AUC, area under ROC curve.

LBC results were evaluated using the Bethesda System, positive threshold were set at ASCUS. NILM means negative and others means positive.

HPV 16/18+: Sensitivity (E6/E7 VS. Cytology): *χ*^2^ = 33.47, *p* < 0.05; Specificity (E6/E7 VS. Cytology): *χ*^2^ = 19.66, *p* < 0.05; PPV (E6/E7 VS. Cytology): *χ*^2^ = 0.017, *p* < 0.05; NPV (E6/E7 VS. Cytology): *χ*^2^ = 13.84, *p* < 0.05.

HPV 16/18−: Sensitivity (E6/E7 VS. 16/18):*χ*^2^ = 0.914, *p* = 0.339; Specificity (E6/E7 VS. 16/18): *χ*^2^ = 44.09, *p* < 0.05; PPV (E6/E7 VS. 16/18): *χ*^2^ = 6.11, *p* = 0.013; NPV (E6/E7 VS. 16/18): *χ*^2^ = 4.29, *p* = 0.038.

AUC (16/18+) *p* < 0.05; AUC (16/18−) *p* < 0.05.

## Discussion

This prospective study establishes HPV E6/E7 ICC assay as a superior triage strategy for HPV-positive women. Unlike assays that only indicate HPV DNA presence, the HPV E6/E7 ICC assay detects a mechanistically grounded biomarker of active oncogenesis, a principle strongly corroborated by key study findings. Furthermore, the assay’s clinical advantage is evidenced by its significantly higher sensitivity for CIN2 + (88.1%) and CIN3 + (88.0%) compared to HPV16/18 genotyping (60.4, 65.6%) and cytology ≥ASC-US (66.2, 75.3%), complemented by an exceptional NPV of 97% for CIN3 + that robustly supports the exclusion of advanced disease. These results confirm that HPV E6/E7 assay effectively identifies transforming infections during the critical transition to HSIL+, addressing a key limitation of current triage methods by more accurately distinguishing women with clinically consequential disease from those with transient, harmless infections.

Despite the implementation of CC screening programs at varying levels under the WHO strategic framework and the significant progress achieved in prevention and control efforts, cervical cancer continues to rank high among gynecological malignancies in both global incidence and mortality rates, with this epidemiological pattern persisting in several countries (23). The HPV test is a useful approach to cervical cancer screening based on the knowledge that 90 ~ 95% of cervical cancer is caused by persistent infection of hrHPV (24). Current triage protocols for cervical hrHPV infections are constrained by suboptimal diagnostic accuracy, often necessitating excessive colposcopies and biopsies to mitigate underdiagnosis risks. This practice not only contributes to inefficient resource allocation within healthcare systems but also introduces potential iatrogenic complications associated with invasive procedures (25). Elevated expression of HPV E6/E7 ICC assay is considered a necessary step for oncogenic transformation of cervical epithelial cells (26). Whereas most previous investigations into HPV E6/E7 ICC assay have been conducted at the nucleic acid level, studies focusing on the protein level itself are comparatively limited, even though the latter may provide more direct functional insights into oncogenesis (27).

Emerging evidence indicates that the clinical performance of the NucliSENS EasyQ HPV v1 assay (bioMérieux, Marcy l’Etoile, France), which detects E6/E7 mRNA from HPV types 16, 18, 31, 33, and 45 using nucleic acid sequence-based amplification (NASBA), is characterized by higher specificity for identifying HSIL+ compared to HPV DNA tests. This enhanced specificity, which helps resolve the sensitivity-specificity dilemma of DNA-based assays, comes at the cost of moderately lower sensitivity (28–32). Nevertheless, a direct comparison between HPV16/18 E6 protein and mRNA detection methods is lacking. Further evaluations are crucial to advance precise screening approaches. Preliminary clinical evaluations indicate that the HPV E6 oncoprotein assay exhibits superior specificity (98.9%) compared to standard HPV DNA tests, including HC2 testing, for detecting CIN3 + lesions, although it demonstrates a lower sensitivity (67.3%) (33, 34).

In this study, the HPV E6/E7 ICC assay showed superior performance for triaging hrHPV-positive women: it achieved higher sensitivity (88%) than conventional E6/E7 assays (60.8–75.5%) (35); it also provided better PPV (0.33 vs. 0.29/0.25) and NPV (0.95 vs. 0.86/0.85) compared to hrHPV genotyping and cytology, respectively. The optimization of ICC, particularly through direct visualization of E6 and E7 dual-proteins in cervical exfoliated cells, has substantially reduced analytical interference of cell morphology. This technical advancement helps improve risk stratification and can mitigate unnecessary patient anxiety associated with transient, non-oncogenic HPV infections. In fact, the enhanced cytomorphological discrimination provided by the dual-staining E6/E7 ICC test underpinned its superior triage capability in our data. It detected 83.6% (56/67) of CIN2 + cases missed by cytology (<ASCUS), while maintaining a low false-negative rate of only 9.9% (13/131) in confirmed CIN2 + cases in Table 3. The complementary role of the E6/E7 ICC test to HPV16/18 genotyping was observed in CIN2 + of the hrHPV cases (Table 2). The test was positive in 81.3% of lesions from HPV16/18-negative women, whereas it was negative in only 7.4% of lesions from HPV16/18-positive women, supporting its utility in reducing missed diagnoses. These results suggest that HPV E6/E7 ICC assay has a higher screening performance and can reduce missed diagnosis. At the same time, its negative test results may be an effective means of diverting people infected with hrHPV. Comparative analysis of triage efficacy between HPV E6/E7 ICC assay and conventional cytology in hrHPV-positive cohorts revealed distinct performance advantages for the HPV E6/E7 ICC assay among HPV16/18-positive individuals. Specifically, the molecular test demonstrated superior sensitivity (93, 95% CI: 0.86–0.96) and enhanced NPV (94, 95% CI: 0.89–0.97), indicating robust risk-stratification capacity for identifying HSIL+. HPV16/18 positive people need to be referred to colposcopy immediately (18–20). We try to find a way to further divert these people to reduce unnecessary colposcopy. Our research has found that HPV E6E7 protein testing shows higher sensitivity and NPV than cytology after genotyping testing. The lower FNR of the HPV E6/E7 ICC assay for detecting CIN2 + supports its use for triaging women who test positive for HPV 16/18. Moreover, a negative protein testing result may allow for conservative management without immediate referral for colposcopy or histopathological examination. Previous studies have found that the risk of CIN3 + with HPV16/18 positive and p16/Ki-67 Dual Stain negative is lower than the referral threshold of colposcopy, which can be observed (36, 37). p16, a tumor suppressor regulating cell cycle progression, and Ki-67, a canonical proliferation marker, exhibit mutually exclusive expression patterns in normal cervical epithelium. The paradoxical co-expression of these biomarkers reflects underlying molecular pathogenesis driven by HPV oncogenesis. Specifically, sustained E6/E7 oncoprotein overexpression induces disrupted cell cycle regulation through Rb/E2F pathway dysregulation and telomerase activation, thereby permitting concurrent p16/Ki-67 immunoreactivity (38). This dual-positive phenotype demonstrates strong clinical correlation with either completed HPV genomic integration events or histopathological progression to cervical neoplasia. For detection of CIN2+, the dual stain had similar sensitivity (83.4%, *p* = 0.1), and statistically higher specificity (58.9%, *p <* 0.05), PPV (21.0% vs. 16.6%, *p <* 0.05), and NPV (96.4% vs. 94.2%, *p* = 0.01) compared with cytology ≥ASC-US (39). Our study demonstrated sensitivity and specificity parameters comparable to previous reports. The observed higher NPV may indicates that this immunocytochemistry methodology employing a dual-staining mechanism analogous to p16/Ki-67 protocols may exhibit comparable triage potential for HPV16/18-positive populations, particularly in excluding non-progressive infections. We listed three determinants more likely to explain the results above. Firstly, the persistently high prevalence of HPV-58 and HPV-52 genotype in Chinese epidemiological landscape (40). Then the included women had intermittent or persistent infection for more than 3 years. In precancerous cells, HPV oncoproteins activate cell cycle progression and inhibit apoptosis which is a process essential for squamous epithelial renewal and tumor prevention (41, 42). These premalignant lesions retain many normal cellular functions, including contact inhibition where non-neoplastic cells stop proliferating upon contact with the basement membrane (43). While such lesions may regress spontaneously, persistent growth typically manifests as concentric expansion that often remains non-invasive for years (41). The limitation of cross-sectional study design leads to the detection results at a certain time point cannot well reflect the whole process of cervical squamous epithelial renewal. Finally, protocol-driven classification of all HPV16/18 and other HPV type coinfections into the HPV16/18 + analytic cohort, consistent with colposcopy triage guidelines for secondary prevention standardization.

In previous studies, few cases of detecting HPV E6/E7 oncoprotein in cervical exfoliated cells by immunocytochemical staining have been retrieved. At present, we have only made a preliminary exploration of cross-sectional design, which has certain limitations. In the future, we look forward to discussing the plan or existing data of longitudinal follow-up to evaluate the predicted value and long-term clinical results. We also look forward that there will be more strategies to improve specificity or combine E6/E7 testing with other markers (e.g., p16/Ki-67 dual staying) to optimize triad performance and reduce false positives in the future. In this research we want to select people who have HPV infection but have not expressed HPV E6/E7 protein abnormally through this method, in order to look forward to conservative observation, rather than immediately referring them to colposcopy. Since all samples were derived from patients undergoing colposcopy and histopathological assessment, the cohort has an enriched prevalence of CIN2 + compared to a true screening population. This spectrum or referral bias likely leads to overestimates of diagnostic sensitivity and AUC. It’s a pity that our experiment is only a diagnostic experiment with exploratory single-center designed, and we did not make a comprehensive analysis of the risk factors. We also hope the cross-sectional nature of the study, and the generalizability of findings given the specific HPV genotype distribution in China. In addition, in order to solve the problem of how to apply it to other populations and environments, especially considering the variation of human papillomavirus genotype distribution, high-quality international multi-center research should attract researchers’ attention.

## Conclusion

In conclusion, the present study provided evidence that HPV E6/E7 ICC assay may be potential new biomarkers for hrHPV positive women. In addition, its performance is better than that of cytology with satisfactory diagnostic values. In the future, a large-scale longitudinal clinical study is needed to evaluate its NPV and triage risk. We hope that this economical and convenient detection method can reduce some unnecessary and traumatic examinations.

## Data Availability

The datasets presented in this article are not readily available because they associated clinical records protected under strict privacy regulations by the originating healthcare institutions. Requests to access the datasets should be directed to jinweimiao@ccmu.edu.cn.
